# Supplementary material for: The synthetic NLR RGA5HMA5 requires multiple interfaces within and outside the integrated domain for effector recognition
Source: Nat Commun. 2024 Feb 6;15:1104. doi: 10.1038/s41467-024-45380-2 (PMC10847126; doi:10.1038/s41467-024-45380-2)
Supplement: Supplementary file 1 — Supplementary Information [file 41467_2024_45380_MOESM1_ESM.pdf]

Supplementary Information for

The synthetic NLR RGA5HMA5 requires multiple interfaces within and outside the integrated domain for effector recognition

Xin Zhang[1,2,5], Yang Liu[1,3,5], Guixin Yuan[1,2], Shiwei Wang[1,2], Dongli Wang[1,3], Tongtong Zhu[1,3], Xuefeng Wu[1,3], Mengqi Ma[1,3], Liwei Guo[1,4], Hailong Guo[1], Vijai Bhadauria[1], Junfeng Liu[1,3,*], You-Liang Peng[1,2,*]

[1]The Ministry of Agriculture Key Laboratory for Crop Pest Monitoring and Green Control, China Agricultural University, Beijing 100193, China.

[2]Frontiers Science Center for Molecular Design Breeding, China Agricultural University, Beijing 100193, China.

[3]Joint International Research Laboratory of Crop Molecular Breeding, China Agricultural University, Beijing 100193, China.

[4]State Key Laboratory for Conservation and Utilization of Bio-Resources in Yunnan, Yunnan Agricultural University, Kunming 650201, China.

[5]These authors contributed equally.

*Correspondence: You-Liang Peng (pengyl@cau.edu.cn), Junfeng Liu (jliu@cau.edu.cn)

Supplementary Figures: 9

Supplementary Tables: 3

Supplementary information

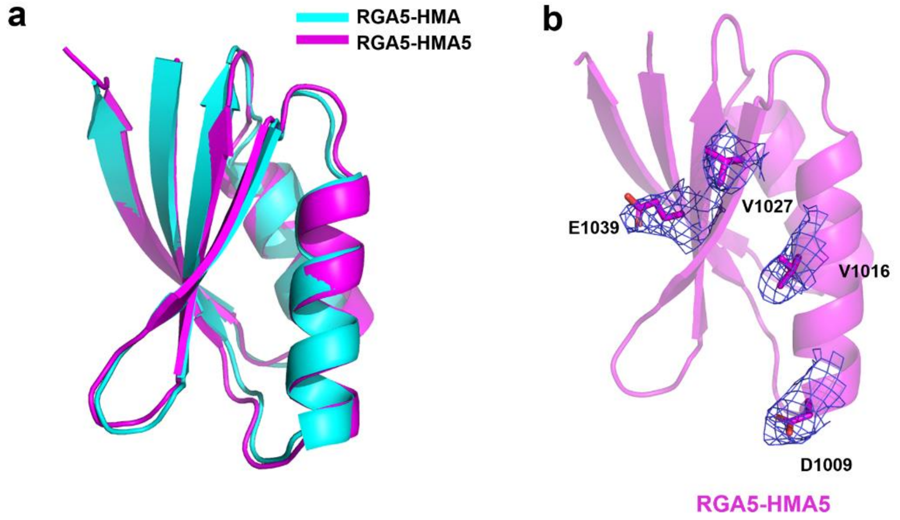

Supplementary Figure 1. Superposition structure of RGA5-HMA and RGA5-HMA5 (a) and the modified residues of the HMA5 domain were shown in blue mesh with the *2mFo-Fc* electron density map contoured at 1.0 σ (b).

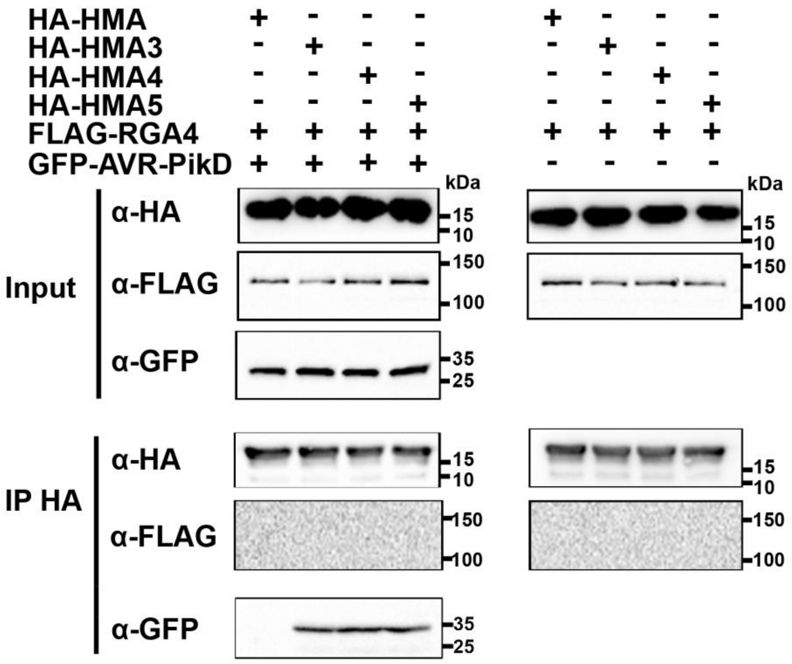

Supplementary Figure 2. Co-IP assays showing that RGA5-HMA and its mutants, RGA5-HMA3, RGA5-HMA4, RGA5-HMA5 do not interact with RGA4 with or without AVR-PikD. Fusion proteins were affinity purified from the infiltrated *N. benthamiana* leaves at 36 h post-infiltration (prior to HR elicitation), followed by immunoblotting using corresponding antibodies. The experiments were repeated twice with similar results.

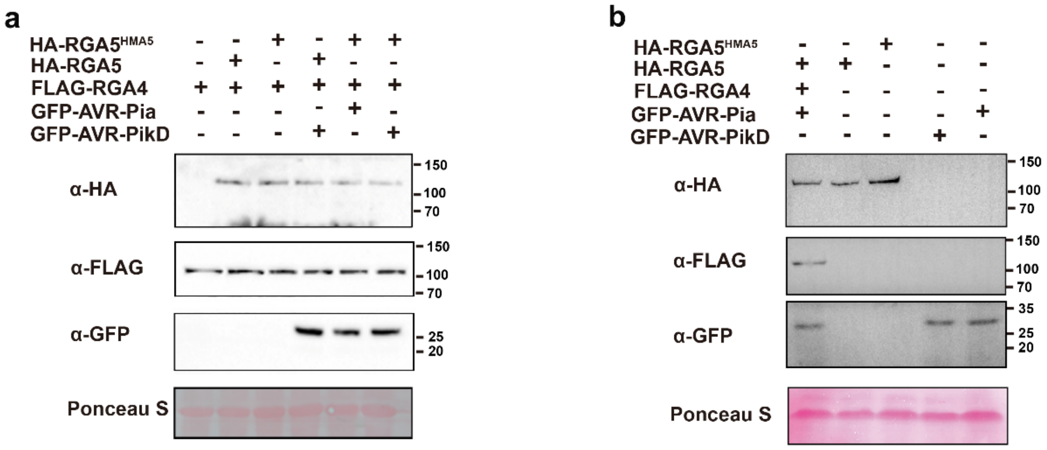

Supplementary Figure 3. Immunoblotting confirming the expression of HA-tagged RGA5 and its mutants with FLAG-tagged RGA4 and GFP-fused effector protein AVR-PikD in different combinations illustrated in Fig.3. Rubisco small submit stained by ponceau S was used to verify equal protein loading. The experiments were repeated twice with similar results.

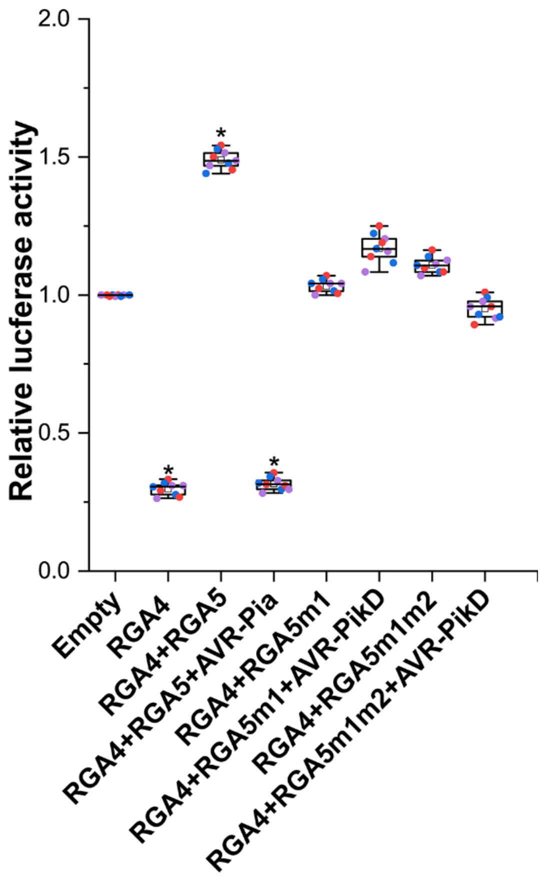

Supplementary Figure 4. The LUC activity of rice protoplasts after transfection with different vector combinations. RGA4 and RGA4/RGA5/AVR-Pia were set as the positive controls, and empty vectors served as the negative control. Significant differences with the empty vector sample are labeled with an asterisk and assessed by Dunnett's HSD test (p <0.05). The assays were repeated three times with similar results.

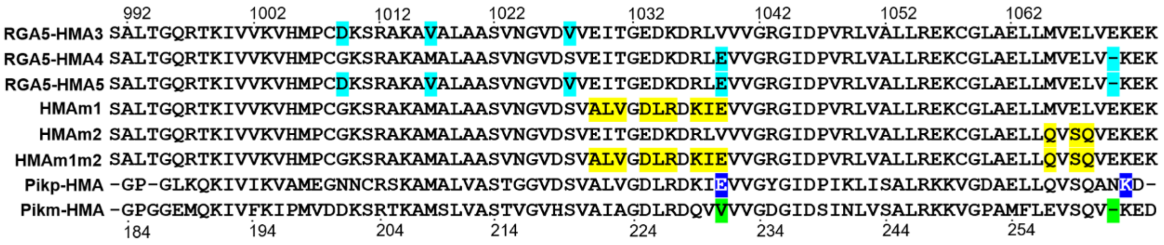

Supplementary Figure 5. The sequence alignment of RGA5-HMA mutants with its mutants and Pikp/Pikm-HMA. The mutations in RGA5-HMA3/4/5 were highlighted in turquoise, and HMAm1/HMAm2/HMAm1m2 in yellow. The key residues involved in the interaction between Piks and AVR-Pik were labeled in blue and green.

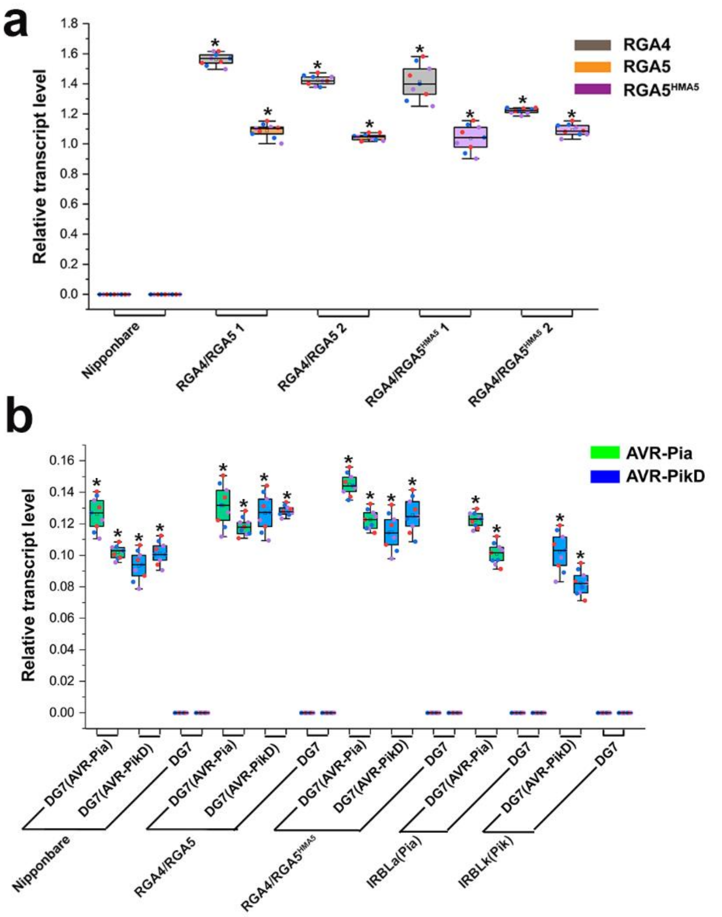

Supplementary Figure 6. RT-qPCR analysis of the expression levels of *RGA4*/*RGA5* and *RGA4*/*RGA5*^{HMA5} in two independent transgenic lines (a) and *AVR-PikD* or *AVR-Pia* after inoculated onto the Nipponbare and transgenic rice leaves corresponding to the combinations in Fig.4 (b). Single asterisks represented significant differences in the expression levels (p <0.05) between wild-type and transgenic lines. The actin gene in rice or the rice blast fungus was used as the internal standard.

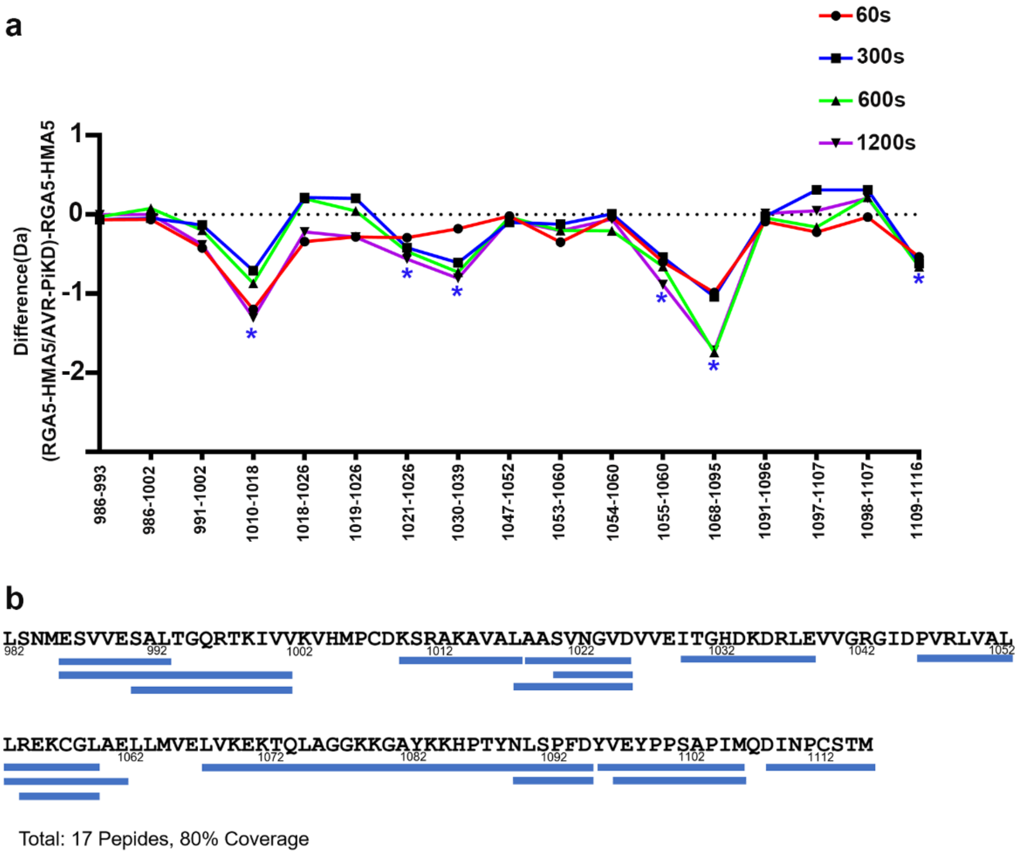

Supplementary Figure 7. HDX-MS analysis of the RGA5-HMA5/AVR-PikD complex. **a** The difference in absolute deuterium uptake between RGA5-HMA5/AVR-PikD and RGA5-HMA5 at different time points (60, 300, 600 and 1200s). Peptides of RGA5-HMA5 in the interaction with AVR-PikD were labeled by asterisks. **b** Sequence coverage in HDX-MS data of AVR-PikD binding sites on RGA5-HMA5.

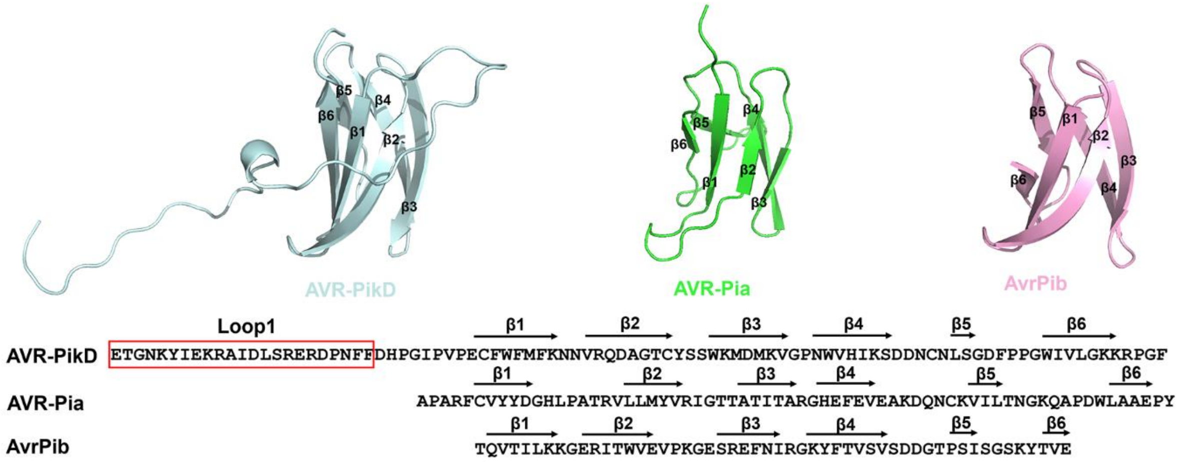

Supplementary Figure 8. The structure and sequence alignment of AVR-PikD with AVR-Pia and AVR-Pib. Loop1 in AVR-PikD was labeled in the red box.

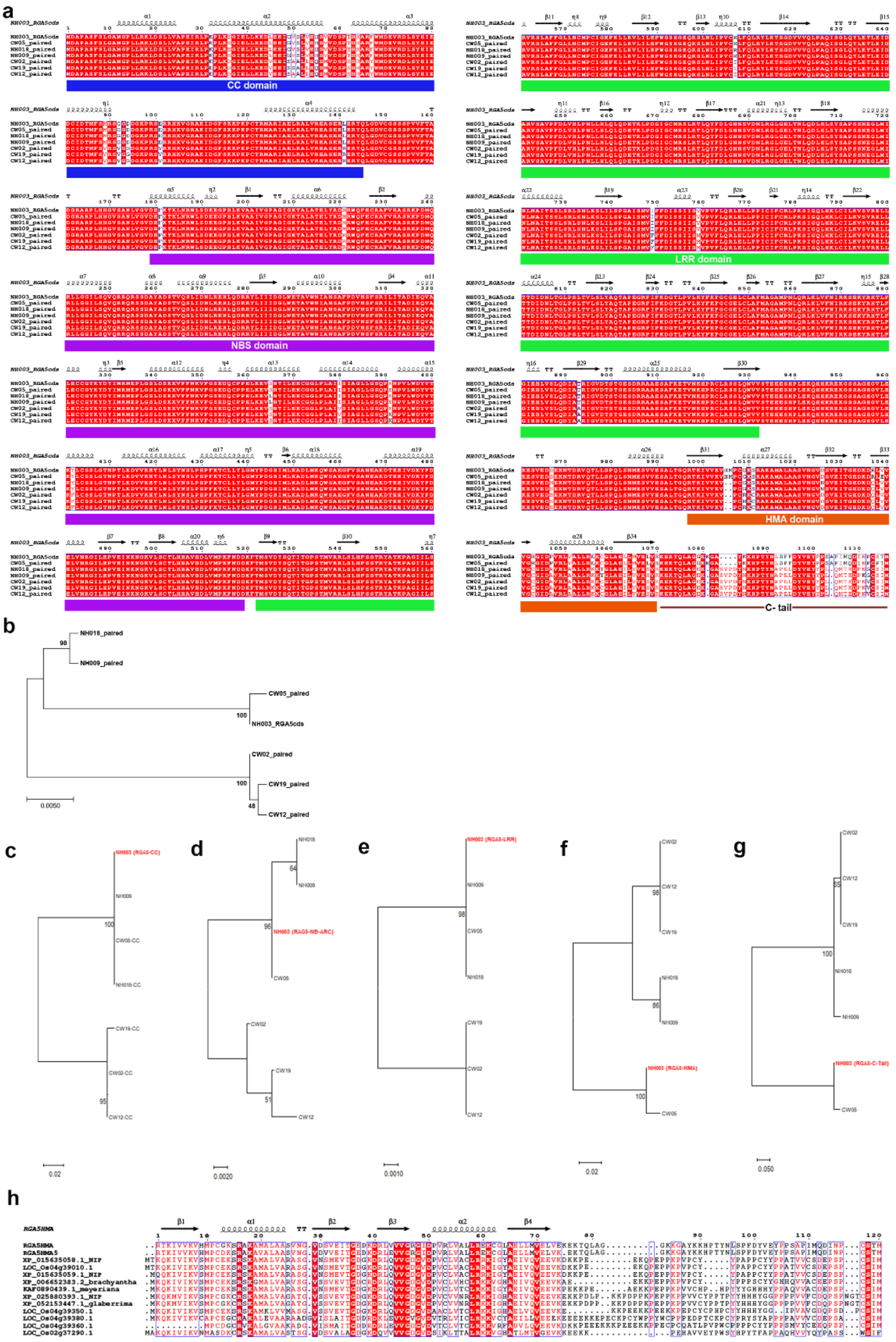

Supplementary Figure 9. Coevolution of RGA5-HMA domain with its C tail. **a** Amino acid sequence alignment of RGA5 orthologs from different rice accessions. NH03 with the wild-type RGA5 is a Geng rice cultivar, while NH09 and NH18 are two Xian rice

cultivars with RGA5 mutations. CW02, CW05, CW12 and CW19 are wild rice. **b** A maximum likelihood-based phylogenetic tree showing the relationship among RGA5 orthologs from rice accessions in (**a**). **c to g** maximum likelihood-based phylogenetic trees showing relationship of RGA5 orthologs in RGA5-CC (**c**), RGA5-NB-ARC (**d**), RGA5-LRR (**e**), RGA5-HMA (**f**) and RHA5-C tail (**g**) domains. The bar length indicates the number of amino acid substitutions per site. **h** Amino acid sequence alignment of rice non-integrated HMA proteins.

Supplementary Table 1. The interactions and phenotypes analysis of the HMA domain or the full-length of RGA5 and mutants with effectors

	RGA5-HMA		RGA5-HMA5	
	AVR-Pia	AVR-PikD	AVR-Pia	AVR-PikD
Y2H	+	-	-	++
Pull down	+	-	(-)	++
MST	+	-	-	++
Co-IP	+	-	(-)	+
CD response in *N. benthamiana*	+	-	-	+
CD in rice protoplasts	+	-	-	+
Effector-triggered immunity in rice	+	-	-	+

Y2H, yeast two-hybrid. MST, microscale thermophoresis. CD, cell death. RGA5-HMA and mutants were used in Y2H, Pull-down and MST assay, the CD assay in *N. benthamiana* and rice protoplasts was carried out by RGA5 and its mutants.

(-), no experimental data; "-", no interaction or no recognition; "+", interaction or recognition, and the number of + indicates the levels of interaction or recognition strength.

Supplementary Table 2. The primers used in this study

Primer code	Sequence (5'-3')	Length
pHAT$_2$-RGA5-HMA F	CATCACCATCACCATCACCTGTCAAACATGGAGAGT	36
pHAT$_2$-RGA5-HMA R	TCTAGAGGGCCCGGATCCTCACATGGTTGAGCAAGG	36
pHAT$_2$-RGA5-HMA5 F	CATCACCATCACCATCACCTGAGCAACATGGAATCC	36
pHAT$_2$-RGA5-HMA5 R	TCTAGAGGGCCCGGATCCCTACATGGTGCTGCACGG	36
pETM13-AVR-Pia F	CTTTAAGAAGGAGATATAGCACCGGCACGTTTTTGT	36
pETM13-AVR-Pia R	GTGGTGGTGGTGGTGGTGCTAATACGGTTCTGCTGC	36
pETSUMO1a-AVR-Pik F	AATCTTTATTTTCAGGGCGAAACCGGCAATAAGTAC	36
pETSUMO1a-AVR-Pik R	GTGGTGGTGGTGGTGGTGTTAGAAACCCGGACGTTT	36
pETMBP1a-HMA F	AATCTTTATTTTCAGGGCCTGTCAAACATGGAGAGT	36
pETMBP1a-HMA R	TCAGTGGTGGTGGTGGTGGTGTCACATGGTTGAGCA	36
pETMBP1a-HMA5 F	AATCTTTATTTTCAGGGCCTGAGCAACATGGAATCC	36
pETMBP1a-HMA5 R	TCAGTGGTGGTGGTGGTGGTGCTACATGGTGCTGCA	36
pCAMBIA 1305-RGA5 F	GGCGCGCCCCTCAGCACGATGGATGCTCCGGCGAGC	36
pCAMBIA 1305-RGA5 R	ATCATGGTCTTTGTAGTCTCACATGGTTGAGCAAGG	36
pCAMBIA 1300-RGA4 F	CAGCTATGACATGATTACATGGAGGCCGCGCTTTTG	36
pCAMBIA 1300-RGA4 R	AAAACGACGGCCAGTGCCTCACTCACAGCAGGAGAC	36
pCAMBIA 1305-RGA5$^{HMA3/4/5}$ F	GGCGCGCCCCTCAGCACGATGGATGCTCCGGCGAGC	36
pCAMBIA 1305-RGA5$^{HMA3/4/5}$ R	ATCATGGTCTTTGTAGTCCTACATGGTGCTGCACGG	36
pCAMBIA 1305-AVR-Pik F	GGCATGGACGAGCTGTACGAAACCGGCAATAAGTAC	36
pCAMBIA 1305-AVR-Pik R	ATCATGGTCTTTGTAGTCTTAGTGGTGGTGGTGGTG	36
pCAMBIA 1305-AVR-Pia F	GGCATGGACGAGCTGTACGCACCGGCACGTTTTTGT	36
pCAMBIA 1305-AVR-Pia R	ATCATGGTCTTTGTAGTCCTAATACGGTTCTGCTGC	36

pKN AVR-PikD F	AAGCTTGATATCGAATTCACGGAGTCTTTAGACGAA	36
pKN AVR-PikD R	TCTCTAGAACTAGTGGATCCCACGTCACCGACGAATTT	38
pKN AVR-Pia F	AAGCTTGATATCGAATTCTTGTTATTTGCTCGCTTA	36
pKN AVR-Pia R	TCTCTAGAACTAGTGGATCCGGCACGTCAGAAGGCTTT	38
pUC19-RGA5 F	GAGAACACGGGGGACGAGATGGATGCTCCGGCGAGC	36
pUC19-RGA5 R	GTCGACCTGCAGGCATGCTCACATGGTTGAGCAAGG	36
pUC19-RGA5$^{HMA3/4/5}$ F	GAGAACACGGGGGACGAGATGGATGCTCCGGCGAGC	36
pUC19-RGA5$^{HMA3/4/5}$ R	GTCGACCTGCAGGCATGCCTACATGGTGCTGCACGG	36
pUC19-AVR-Pia F	GAGAACACGGGGGACGAGGCACCGGCACGTTTTTGT	36
pUC19-AVR-Pia R	GTCGACCTGCAGGCATGCCTAATACGGTTCTGCTGC	36
pUC19-AVR-Pik F	GAGAACACGGGGGACGAGATGGAAACCGGCAATAAG	36
pUC19-AVR-Pik R	GTCGACCTGCAGGCATGCCTAGAAACCCGGACGTTT	36
pUC19-ΔAVR-Pik F	GAGAACACGGGGGACGAGATGCACCCAGGCATTCCAG	37
pUC19-ΔAVR-Pik R	GTCGACCTGCAGGCATGCCTAGAAACCCGGACGTTT	36
pGBKT7-AVR-PikD F	ATCTCAGAGGAGGACCTGGAAACCGGCAATAAGTAC	36
pGBKT7-AVR-PikD R	AAGGGGTTATGCTAGTTAGAAACCCGGACGTTTCTT	36
pGBKT7-ΔAVR-PikD F	ATCTCAGAGGAGGACCTGCACCCAGGCATTCCAGTT	36
pGBKT7-ΔAVR-PikD R	AAGGGGTTATGCTAGTTAGAAACCCGGACGTTTCTT	36
pGBKT7-AVR-Pia F	ATCTCAGAGGAGGACCTGGCACCGGCACGTTTTTGT	36
pGBKT7-AVR-Pia R	AAGGGGTTATGCTAGTTACTAATACGGTTCTGCTGC	36
pGADT7-RGA5-HMA F	GACGTACCAGATTACGCTCTGTCAAACATGGAGAGT	36
pGADT7-RGA5-HMA R	GGGGTTTTTCAGTATCTATCACATGGTTGAGCAAGG	36
pGADT7-RGA5-HMA3/4/5 F	GACGTACCAGATTACGCTCTGAGCAACATGGAATCC	36
pGADT7-RGA5-HMA3/4/5 R	GGGGTTTTTCAGTATCTACTACATGGTGCTGCACGG	36

Supplementary Table 3. Rice Accessions in which RGA5 orthologs were identified and used for generating phylogenetic trees

Accession*	Name	Original area	Species information
NH003	Zaoshengbai	China	*O. sativa japonica*
NH009	Malaihong	Malaysia	*O. sativa indica*
NH018	Buleida A-75	Mexico	*O. sativa indica*
CW02	NA	China	*O. rufipogon*
CW05	NA	Brunei	*O. rufipogon*
CW12	NA	China	*O. rufipogon*
CW19	NA	China	*O. rufipogon*

*The RGA5 orthologs in the rice accessions were reported previously[1].

References

1. Shang, L. et al. A super pan-genomic landscape of rice. *Cell Res.* **32**, 878-896 (2022).
